# Core Versus Vacuum-Assisted Postbiopsy Breast Clip Migration Rates

**DOI:** 10.7759/cureus.84113

**Published:** 2025-05-14

**Authors:** Paul Jaszewski, Hilda Tejero

**Affiliations:** 1 Department of Radiology, Hospital Corporation of America (HCA) Florida Aventura Hospital, Aventura, USA; 2 Department of Radiology, Hospital Corporation of America (HCA) Florida Mercy Hospital, Miami, USA; 3 Department of Radiology, Hospital Corporation of America (HCA) Florida Kendall Hospital, Miami, USA

**Keywords:** breast cancer care, breast cancer management, breast cancer treatment, clip migration, surgical clip migration

## Abstract

Background: Biopsy clip migration is a well-known complication that can occur after breast biopsies with potentially devastating effects on patient care and treatment. The purpose of this study is to compare the rates of postbiopsy clip migration after the two most common methods of biopsy: core-needle biopsy (Core) and vacuum-assisted biopsy (VAB).

Methodology: A retrospective review of 153 postbiopsy mammograms (103 Core and 50 VAB) was conducted from March 2024 to March 2025 at three breast imaging centers in southeastern Florida. Only postbiopsy mammograms from a single, randomly selected radiologist will be evaluated to serve as a self-control for any differences in techniques between radiologists. Mammographically occult lesions were excluded from evaluation. Rates of clip migration (with clinically significant migration being defined as 5 mm or greater) were evaluated between the two biopsy techniques while controlling for age and breast density (as defined by Breast Imaging Reporting and Data System nomenclature).

Results: Of the 153 biopsies examined, 18 (11.76%) demonstrated clinically significant clip migration (≥5 mm). Of these, VAB demonstrated a significantly higher migration rate of 24% (12 cases) compared to Core biopsies (5.8%, six cases) (p = 0.00265).

Conclusions: The findings indicate that VAB is associated with a significantly increased risk of clip migration compared to Core biopsies. Optimization of clip deployment strategies in VAB procedures may mitigate migration risk. Further prospective studies are warranted to refine procedural techniques, materials and enhance clip stability to improve localization accuracy in subsequent interventions.

## Introduction

Breast cancer remains a leading cause of oncologic mortality worldwide, being the second most common cancer overall, the most common cancer in women, and the most common cancer mortality among women [1]. In response to the prevalence of breast cancer, newer and more advanced forms of percutaneous tissue sampling have been developed over time, leading to these percutaneous techniques becoming standard of care for the evaluation of breast pathologies [2]. According to a 2020 analysis of Medicare Part B, imaging-guided percutaneous biopsies increased 51% over the span of 2004-2016 (from 124,423 to 187,914) vs. a decrease in open surgical breast biopsies by 64% over the same span (from 6,605 to 2,373) [2]. There are several reasons for this shift from surgical biopsies to percutaneous biopsy techniques, including their minimally invasive nature, decreased complication rates, and their quick recovery times while maintaining diagnostic accuracy and reliability [2,3].

While multiple techniques are available for percutaneous biopsies, the current two main methods are core-needle biopsies (Core) vs. vacuum-assisted biopsies (VAB), both with their own advantages and disadvantages [3]. Regardless of the type of percutaneous biopsy performed, placing a biopsy clip/marker at the biopsy site has become standard of care [4]. Immediate placement of a biopsy marker allows quick and accurate localization of the breast biopsy location, which is crucial for guiding subsequent treatments such as surgery, radiotherapy, or monitoring [4]. In one study from 2015, lack of breast clip placement post biopsy demonstrated a relative risk of local reoccurrence of 4.8, highlighting the importance of tumor bed markers for adequate follow-up and treatment [4].

One of the most well-known complications of breast biopsy clip placement is known as clip migration, which refers to the unintended movement of the marker clip from its original placement site within breast tissue. Migration can lead to unnecessary repeat biopsies, incorrect preoperative localization, and increased patient morbidity [5].

Several factors have been suggested to cause this phenomenon, such as breast composition, breast thickness, lesion location, biopsy technique, breast compression (the so-called “Accordion effect”), biopsy tract position, hematoma formation, and even the types of clips themselves, to name a few [5-10]. Very few reports, however, have directly studied the relationship between clip migration rates and the two most common types of biopsy procedures: core-needle biopsy (Core) and VAB. Therefore, this study aims to compare clip migration rates between VAB and Core biopsies.

## Materials and methods

Study design

This study is a single-radiologist, retrospective review conducted within the Hospital Corporation of America (HCA) East Florida Division hospitals (HCA Florida Aventura, HCA Florida Kendall, and HCA Florida Mercy). It aimed to evaluate the rate and degree of breast clip migration following two common types of percutaneous breast biopsy procedures: Core and VAB. The single radiologist was randomly selected and had performed both biopsy techniques within the study period (from March 2024 to March 2025), serving as an internal control to reduce interoperator variability.

Ethical considerations

This retrospective study was reviewed and determined to meet criteria for exemption from full Institutional Review Board oversight under HCA institutional guidelines. No protected health information or identifiable data were collected. All data were fully deidentified and stored in a Health Insurance Portability and Accountability Act-compliant manner within the secure HCA Healthcare network. The identity of the selected radiologist remained confidential and accessible only to the research team.

Study criteria

Eligible cases included female patients who underwent either Core or VAB performed by the selected radiologist during the study period, with available postbiopsy mammograms showing visible lesions and clips. Exclusion criteria included biopsies performed by other radiologists, absence of postbiopsy imaging, or mammographically occult lesions. After initially identifying and screening 227 potential cases, one case was excluded due to missing postbiopsy imaging, and 73 other cases were excluded for having mammographically occult lesions, leaving 153 eligible cases (103 Core and 50 VAB).

Procedure

Postbiopsy mammograms were reviewed for each included case. The largest migration distance between the biopsy clip and the target lesion was recorded, using the greatest value from craniocaudal, mediolateral oblique, or mediolateral views. Migration distance was measured in millimeters, with clinically significant migration defined as a displacement of ≥5 mm.

Assessments

Data collected included biopsy type (Core vs. VAB), patient age, and breast density. Breast density was categorized using the Breast Imaging Reporting and Data System Fifth Edition guidelines and nomenclature (Entirely Fatty also known as Category a, Scattered Fibroglandular also known as Category b, Heterogeneously Dense also known as Category c, and Extremely Dense also known as Category d).

The density classification was determined by an initial impression from a primary reviewer. This was then cross-referenced with the original mammogram report. In cases of breast density determination discrepancy, a second reviewer provided an assessment, and the majority consensus was used.

Sample size calculation

A sample size calculation was performed to estimate the number of participants required to detect a statistically significant difference in clinically significant clip migration between the Core and VAB groups. Based on expected migration rates of 25% in the VAB group and 5% in the Core group, with a 2:1 allocation favoring the Core group, a two-sided alpha of 0.05, and 80% power, the estimated minimum required sample size was 84 participants (56 Core and 28 VAB).

A post hoc power analysis using the actual group sizes (103 Core and 50 VAB) and observed clinically significant migration rates of 5.8% and 24.0%, respectively, yielded a calculated statistical power of 87.1%, confirming that the study was adequately powered to detect the observed effect.

Statistical analysis

Descriptive statistics were used to summarize clip migration distances, age, and breast density. Welch’s t-test was used to compare mean migration distances between the Core and VAB groups. The chi-square test assessed the association between biopsy type and the frequency of clinically significant migration (≥5 mm). Cohen’s d was calculated to measure the effect size between the groups. One-way analysis of variance (ANOVA) was used to analyze variations in migration distance across breast density categories. Pearson correlation was employed to explore associations between patient age and clip migration. Fisher’s exact test (two-tailed) was used to compare clip migration rates between the Core and VAB biopsy types in breast density subgroups. Statistical significance was set at p < 0.05. All analyses were conducted using STATA statistical software (StataCorp, College Station, TX).

## Results

One hundred fifty-three postbiopsy mammograms were analyzed, all of which were of women (100%). The mean age of all patients was 59.76, ranging from 25 to 97. Of the 153 postbiopsy mammograms identified, 50 were noted to have undergone VAB while 103 underwent Core. Between the Core and VAB groups, there was no significant difference in average age (59.45 vs. 60.42, p = 0.658). The most common breast density was Heterogeneously Dense with 57 patients (37.25%), followed by Scattered Fibroglandular with 52 patients (33.99%), Entirely Fatty with 24 patients (15.69%), and finally Extremely Dense with 20 patients (13.07%). Between the Core and VAB groups, chi-square analysis demonstrated that there was no significant difference in patient breast density (p = 0.281) (Table 1).

**Table 1:** Sociodemographic and baseline patient characteristics Breast density categorizations as per ACR BI-RADS Fifth Edition: Extremely dense: the breasts are extremely dense, which lowers mammographic sensitivity also known as Category d; Entirely fatty: the breasts are almost entirely fatty also known as Category a; Scattered fibroglandular: there are scattered areas of fibroglandular density also known as Category b; Heterogeneously dense: the breasts are heterogeneously dense, which may obscure small masses also known as Category c Core: core-needle biopsy; VAB: vacuum-assisted biopsy; ACR BI-RADS: American College of Radiology Breast Imaging-Reporting and Data System

Characteristic	Core	VAB	Total (n = 153)	Test statistic	p value
Number of patients (n)	103	50	153	-	-
Mean age (years)	59.45 (±15.32)	60.42 (±11.28)	59.76 (±14.10)	t = -0.443	0.658
Breast density					
Extremely dense	15 (14.56%)	5 (10%)	20 (13.07%)	χ² = 3.82	0.281
Entirely fatty	13 (12.62%)	11 (22%)	24 (15.69%)		
Scattered fibroglandular	33 (32.04%)	19 (38%)	52 (33.99%)		
Heterogeneously dense	42 (40.78%)	15 (30%)	57 (37.25%)		

Of the 153 postbiopsy mammograms identified, 50 were noted to have undergone VAB while 103 underwent Core. The average migration distance (in millimeters) was 1.23 ± 1.9 for Core biopsies and 3.52 ± 3.58 for VAB. The 95% confidence intervals (CIs) for Core biopsies were 0.87-1.60 vs. 2.53-4.51 mm for VAB, suggesting a significant difference as the CIs for Core and VAB do not overlap. Welch’s t-test showed a statistically significant difference in mean migration between these groups (p = 0.00007). Of the 103 Core biopsies, six (5.8%) demonstrated clinically significant clip migration (5 mm or greater). Of the 50 VAB, 12 (24.0%) demonstrated clinically significant clip migration. Chi-square showed a statistically significant difference in clinically significant migration rates between these groups (p = 0.00265) (Table 2). Calculation of effect size (Cohen’s d) yielded -0.80, indicating a large effect size, which supports that the difference in clip migration between Core and VAB is substantial.

**Table 2:** Mean migration distance and clinically significant migration rates for core vs. VAB Core: core-needle biopsy; VAB: vacuum-assisted biopsy

Parameter	Core	VAB	Test statistic	p value
Average migration distance (mm)	1.23 (±1.9)	3.52 (±3.58)	t = -4.24	0.00007
Clinically significant migration rate, n (%)	6 (5.8%)	12 (24%)	χ² = 9.03	0.00265

There was no significant correlation between patient age and either mean clip distance (millimeters) (p = 0.9076) or clinically significant migration rate (p = 0.7801) (Table 3).

**Table 3:** Correlation coefficients of age versus mean clip migration distance and clinically significant migration rate

Comparison	Correlation coefficient	p value
Age vs. mean clip migration distance	0.0095	0.9076
Age vs. clinically significant migration rate	-0.0228	0.7801

**Figure 1:**
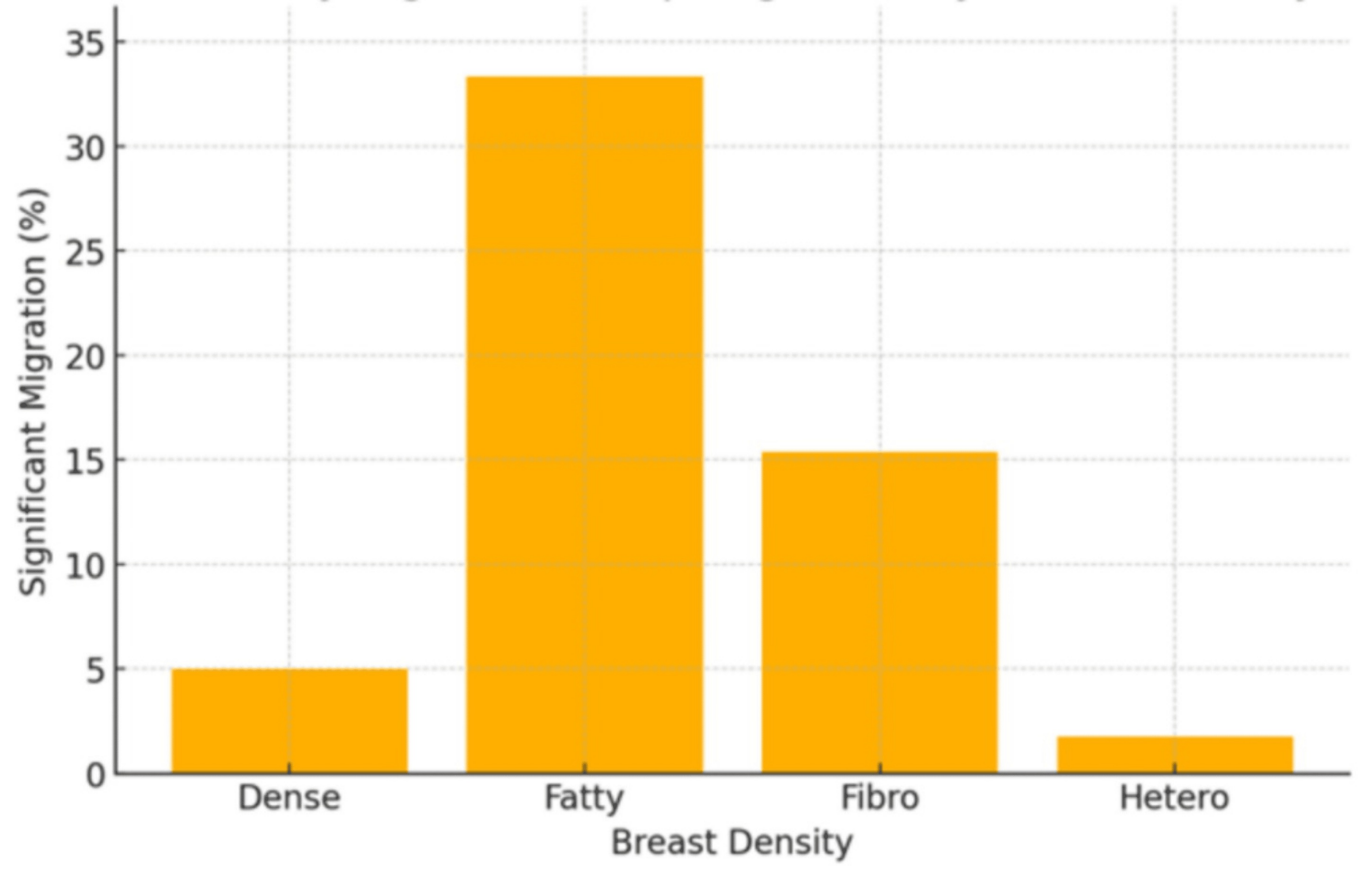
Rate of clinically significant breast clip migration based on breast density

In terms of breast density, Entirely Fatty breasts had the highest average migration distance at 4.21 mm, followed by Scattered Fibroglandular at 1.65 mm, Heterogeneously Dense at 1.51 mm, and finally Extremely Dense at 1.5 mm, with ANOVA demonstrating a statistically significant difference in mean clip migration distance between different breast densities (p = 0.00023). In terms of clinically significant clip migration rates, Entirely Fatty breasts had the highest rate at 33.3% (eight cases), followed by Scattered Fibroglandular at 15.38% (eight cases), Extremely Dense at 5.0% (one case), and finally Heterogeneously Dense at 1.75% (one case), with chi-square analysis demonstrating statistically significant differences in rates between different breast densities (p = 0.00048) (Table 4, Figure 1).

**Table 4:** Mean migration distance and clinically significant migration rates for different breast densities Breast density categorizations as per ACR BI-RADS Fifth Edition: Extremely dense: the breasts are extremely dense, which lowers mammographic sensitivity also known as Category d; Entirely fatty: the breasts are almost entirely fatty also known as Category a; Scattered fibroglandular: there are scattered areas of fibroglandular density also known as Category b; Heterogeneously dense: the breasts are heterogeneously dense, which may obscure small masses also known as Category c ACR BI-RADS: American College of Radiology Breast Imaging-Reporting and Data System

Parameter	Extremely dense	Entirely fatty	Scattered fibroglandular	Heterogeneously dense	Test statistic	p value
Average clip migration (mm)	1.5	4.21	1.65	1.51	F = 6.85	0.00023
Number and rate of clinically significant migrations	1 (5%)	8 (33.33%)	8 (15.38%)	1 (1.75%)	χ² = 17.80	0.00048

**Figure 2:**
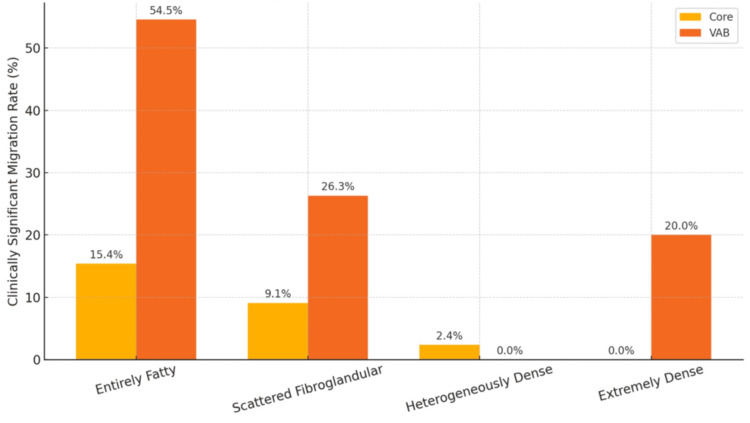
Rate of clinically significant clip migration by biopsy type and breast density

Breast density subgroup analysis revealed that the greatest difference between Core and VAB clinically significant migration rates was found in Entirely Fatty breasts (15.38% vs. 54.55%, p = 0.0825). In Scattered Fibroglandular breasts, Core and VAB showed migration rates of 9.09% and 26.32%, respectively (p = 0.1241). Heterogeneously Dense and Extremely Dense cohorts showed minimal differences in migration rates between Core and VAB, with p values of 1.0000 and 0.2500, respectively (Tables 5, 6 and Figure 2). No statistical significance was noted in any of the subgroup comparisons.

**Table 5:** Number of clinically significant migrations based on breast density and biopsy type Breast density categorizations as per ACR BI-RADS Fifth Edition: Extremely dense: the breasts are extremely dense, which lowers mammographic sensitivity also known as Category d; Entirely fatty: the breasts are almost entirely fatty also known as Category a; Scattered fibroglandular: there are scattered areas of fibroglandular density also known as Category b; Heterogeneously dense: the breasts are heterogeneously dense, which may obscure small masses also known as Category c Core: core-needle biopsy; VAB: vacuum-assisted biopsy; ACR BI-RADS: American College of Radiology Breast Imaging-Reporting and Data System

Breast density	Biopsy type	Total cases	Clinically significant migrations
Entirely fatty	Core	13	2
	VAB	11	6
Scattered fibroglandular	Core	33	3
	VAB	19	5
Heterogeneously dense	Core	42	1
	VAB	15	0
Extremely dense	Core	15	0
	VAB	5	1

**Table 6:** Clinically significant migration rates based on breast density and biopsy type Breast density categorizations as per ACR BI-RADS Fifth Edition: Extremely dense: the breasts are extremely dense, which lowers mammographic sensitivity also known as Category d; Entirely fatty: the breasts are almost entirely fatty also known as Category a; Scattered fibroglandular: there are scattered areas of fibroglandular density also known as Category b; Heterogeneously dense: the breasts are heterogeneously dense, which may obscure small masses also known as Category c Core: core-needle biopsy; VAB: vacuum-assisted biopsy; ACR BI-RADS: American College of Radiology Breast Imaging-Reporting and Data System

Breast density	Core clinically significant migration rate (%)	VAB clinically significant migration rate (%)	Fisher exact p value
Entirely fatty	15.38	54.55	0.0825
Scattered fibroglandular	9.09	26.32	0.1241
Heterogeneously dense	2.38	0	1
Extremely dense	0	20	0.25

## Discussion

This risk of clip displacement has potentially serious short- and long-term repercussions for both the patient and their providers, including necessitating rebiopsy or potential need for additional or greatly expanded surgical intervention [5,9,11,12]. In this current study, it was found that 24% of all VABs demonstrate 5 mm or greater clip migration compared to 5.8% of Core biopsies. This suggests that VAB is associated with significantly higher clip migration rates than Core biopsy, both statistically and clinically. Additionally, this study suggested that there is a meaningful correlation between breast density and breast clip migration, particularly as breast density decreases, which correlates with multiple previous research studies [6]. No significant correlation between breast clip migration and age was identified.

The substantial difference in migration rates between the two most common biopsy techniques suggests that vacuum assistance may contribute to increased clip displacement. This has been hypothesized to potentially be due to the larger volume of tissue removal and mechanical forces exerted during the procedure, as suggested in previous studies [6-10]. Future studies comparing the volume of tissue sample removed to clip migration rates could help determine if a correlation exists.

Furthermore, the higher rate of clip migration in Entirely Fatty breasts (33.3%) supports the idea that reduced tissue density contributes to reduced clip stability. Radiologists should be particularly cautious when performing VAB in patients with fatty breasts, as this combination appears most prone to clinically significant displacement.

Breast density subgroup analysis supports this concern. Entirely Fatty breasts demonstrated a large difference in clinically significant migration rates between VAB (54.55%) and Core (15.38%), as did Scattered Fibroglandular breasts (VAB 26.32% vs. Core 9.09%); however, it should be noted that none of the breast density subgroups demonstrated statistical significance when it came to clinically significant migration rates vs. biopsy type, which may be due to the small subgroup sample sizes. This may be a worthy target for future larger, prospective investigations.

The overall increased migration observed in VAB procedures underscores the necessity for improved clip deployment methodologies to avoid the potentially devastating effects of clip migration. Previous studies have primarily focused on the potential effects of patient positioning, particularly with a lateral arm approach, with mixed results [6,13]. Another study evaluated the usage of breast-specific biopsy clips with positive results and theorized that the outer component of the breast clips acts as an anchor to minimize migration [10]. In comparison, this study demonstrates the role of the biopsy method in potentially causing the clip migration. Further future research into the technical components of VAB guns and materials, such as gauge size, percentage of sample removed, or needle design, with relation to clip migration, seems prudent.

Several limitations of this study are present. First, there was variation in the types of clips placed and the biopsy equipment used. Ideally, clip type would be controlled for to eliminate potential bias among types of biopsy clips; however, all of the biopsy clips utilized were breast-biopsy-specific clips. Second, this study did not separate biopsies based on ultrasound vs. stereotactic-guided. Core-needle biopsies are predominantly done under ultrasound guidance, while vacuum-assisted biopsies are commonly done under both ultrasound and stereotactic guidance. It would be prudent to further evaluate whether the stereotactic vs. ultrasound-guided technique provides different rates of clip migration.

## Conclusions

This study has shown that there is a significantly higher correlation between breast clip migration and vacuum-assisted biopsies (VAB, 24%) relative to core-needle biopsies (Core, 5.8%). Additionally, this study further supported the relationship between lower breast tissue density and increased clip migration rates. Future studies into the materials and methods of vacuum-assisted biopsies to potentially reduce the rate of clip migration are considered worthwhile due to the potentially negative devastating effects.
